# Obstetric anesthesia clinic childbirth course combined with labor epidural analgesia is associated with a decreased risk of postpartum depression : a prospective cohort study

**DOI:** 10.1186/s12871-022-01931-y

**Published:** 2022-12-15

**Authors:** Shanshan Tong, Chuanhua Rao, Su Min, Hua Li, Dongqun Quan, Daping Chen, Yuanmao Zhu

**Affiliations:** 1 Department of Anesthesiology, The First Affiliated Hospital of Chongqing Medical University, No.1 Youyi Road, Yuzhong District, Chongqing, 400016 People’s Republic of China; 2 Department of Anesthesiology, Jiangjin Central Hospital of Chongqing, No.725 Jiangzhou Avenue, Dingshan Street, Jiangjin District, Chongqing, China; 3 Department of Obstetrics, Jiangjin Central Hospital of Chongqing, Chongqing, China; 4 Department of Pain, Jiangjin Central Hospital of Chongqing, Chongqing, China

**Keywords:** Postpartum depression (PPD), Labor epidural analgesia, Childbirth course, Edinburgh postpartum depression scale (EPDS)

## Abstract

**Background:**

Postpartum depression (PPD) is a serious complication commonly seen in postnatal women. In this paper, an investigation was conducted to see if obstetric anesthesia clinic childbirth course combined with labor epidural analgesia (LEA) was associated with a decreased risk of PPD.

**Methods:**

Six hundred fifty-five nulliparous women were enrolled in this prospective cohort study. The parturients were divided into 4 groups, with Group C being the control group, Group AC received the obstetric anesthesia clinic childbirth course only, Group LEA received LEA only, and Group AC + LEA received both the obstetric anesthesia clinic childbirth course and LEA. Maternal and neonatal variables in the perinatal period were recorded. PPD at 6 weeks was assessed using the Chinese version of the Edinburgh Postpartum Depression Scale (EPDS), where a score ≥ 10 is the threshold for PPD. Multivariate logistic regression analysis was performed to assess the association between obstetric anesthesia clinic childbirth course combined with LEA and postpartum depression.

**Results:**

A total of 124 maternities had EPDS ≥10 points, the incidence of PPD was 18.9%。The incidence of PPD and EPDS scores were significantly lower in Group AC + LEA than in Group C (12.1% vs 26.8%, *P* <  0.05; 6 (5, 7) vs 7 (5, 11), *P* <  0.05). Received an anesthesia clinic childbirth course combined with LEA was associated with a decreased risk of PPD (OR 0.273, 95% CI, 0.100–0.743, *P* = 0.013). Multivariate logistic regression analysis identified 5 other independent factors for PPD, including maternal SAS score in the delivery room, W-DEQ score in the delivery room, living in a confinement center, EPDS score at 1st week postpartum and perinatal care satisfaction .

**Conclusions:**

Received an obstetrics anesthesia clinic childbirth course combined with LEA for nulliparous women with a single term cephalic pregnancy was associated with a decreased risk of PPD at 6 weeks.

**Trial registration:**

Chinese Clinical Trial Registry, ChiCTR2000039163. Registered on 20/10/2020.

## Introduction

Postpartum depression (PPD) is defined in the Diagnostic and Statistical Manual for Mental Disorders as a major depression with postpartum onset with episodes of depression beginning within 4 weeks of giving birth [1–4]. PPD poses significant risks to mothers and their children, including the risk of maternal suicide, infanticide, infant growth retardation and reduced maternal-infant attachment [5–8]. Several risk factors contributing to PPD have been identified, such as psychosocial stressors, family and spousal support, income, and marriage [9–13]. Labor pain is one of the most serious causes of pain and distress in a woman’s life, with approximately 60% of first-time mothers describing their pain as severe or extremely severe [14–16], and it is associated with increased risks of episodes of psychiatric disorders [17–19]. Epidural analgesia is the gold standard for pain relief during labor [20]. Several studies have found that the use of labor epidural analgesia (LEA) during childbirth is associated with decreased rates of PPD at 6 weeks postpartum [21–23]. In contrast some studies have shown no effect of labor epidural analgesia on postpartum depression [24, 25], while another study showed a harmful effect for women who planned to avoid, but ended up using labor epidural analgesia [26]. As prenatal anxiety, fear of childbirth and unbearable labor pain are the most important reasons why women choose cesarean section without any medical reason [27–30]. Antenatal anxiety and fear of childbirth are associated with the development of PPD [31]. Childbirth courses may reduce pregnant women’s anxiety and fear [32, 33]. At our hospital, routine prenatal care is limited to regular check-ups, and ultrasounds. The curriculum of childbirth courses does not include relevant knowledge of LEA and perinatal psychiatric disorders, and the prenatal education team does not have a specialist anaesthetist. Our hypothesis is that received an obstetrics anesthesia clinic childbirth course and LEA would be associated with a lower risk for PPD.

## Methods

### Patient recruitment

The research activity was approved by the Ethics Committee of the First Affiliated Hospital of Chongqing Medical University (KY2020–598), and was registered with Chinese Clinical Trial Registry (ChiCTR20000391 63). Written informed consent was obtained from all participants before collecting their data, and the study was conducted in accordance with the Declaration of Helsinki.

Potential participants were screened at anaesthesia clinic at 36–40 weeks of gestation. Inclusion criteria were nulliparas with term singleton cephalic pregnancy, who were planning to come to our hospital for vaginal delivery, had a primary school education or above, were able to accurately understand the scale and questionnaire contents, and voluntarily participated in this research. Exclusion criteria included adolescent mothers (age < 18 years), a history of psychiatric diseases, a score of ≥10 on the Edinburgh Postnatal Depression Scale (EPDS), presence of contraindications for labor epidural analgesia. Rejection criteria was the conversion of vaginal delivery to cesarean section.

### The obstetric anaesthesia clinic childbirth course

Before the obstetric anesthesia clinic was open, the midwife first informed the pregnant woman that the anesthesiology department can provide LEA services when the pregnant woman entered the delivery room. After the pregnant woman chose LEA according to her wishes, the midwife informs the anesthesiologist that there are pregnant women who wish to use LEA. Then the anesthesiologist evaluated the pregnant woman for LEA, and began LEA for the woman.

In order to improve the safety of maternal anesthesia and the service ability of anesthesiologists, and to reduce the rush of pregnant women between outpatient clinics, an obstetric anesthesia clinic was set up in the obstetric outpatient clinic of our hospital, so that pregnant women were more willing to accept the services of the obstetric anesthesia clinic. The obstetric anesthesia clinic operates three times a week. The main function was to provide childbirth courses for pregnant women of 36–40 w pregnancy and to evaluate and guide pregnant women with complications and complications during pregnancy, so as to prevent or reduce postpartum complications.

The obstetric anesthesia outpatient delivery course is designed and implemented under the guidance of the psychiatrist in our hospital. It is taught by anesthesiologists and anesthesia nurses in the research group. The course content was as follows.

1. Anesthesiologists publicize LEA, mainly introducing the indications, contraindications, safety, reliability, complications, implementation process, precautions, experience sharing and cost of puerperae who have received LEA.
2. Pregnant women with prenatal depression were screened by EPDS score.
3. Anesthesiologists introduced knowledge about prenatal anxiety, fear of childbirth and perinatal depression.
4. Anesthesiologists introduced the environment of the delivery room through video to reduce the fear and strangeness of pregnant women when entering the delivery room.

### Grouping

All eligible parturients were informed about the research, and consent was obtained for study participation. There were four groups, and the decision whether to receive LEA was made by each woman herself. Before the opening of the obstetric anesthesia clinic, some women were divided into the Group C (neither obstetric anesthesia clinic childbirth course nor LEA) and the Group LEA (only LEA). After the obstetric anesthesia clinic was opened, other women participated in the childbirth course, and then they were divided into Group AC (only obstetric anaesthesia clinic childbirth course) and Group AC + LEA (both obstetric anesthesia clinic childbirth course and LEA).

### Labor epidural analgesia

For parturients who received LEA, LEA was initiated when the cervix was dilated to 1 cm or more. For women who did not require LEA, no analgesia was administered. Epidural space puncture and catheterization was performed at L2-L3 interspace, and 5 ml of 1.2% lidocaine plus 1:200,000 epinephrine was given as a test dose. After 5 minutes, 10 ml of 0.1% ropivacaine plus 0.5 μg/ml sufentanil was administered as a loading dose. An additional dose of 5 ml mixture was administered 10 minutes later if the numeric rating scale (NRS, an 11-point scale where 0 = no pain and 10 = worst pain) of pain was above 4. If the NRS score remained above 4 after the supplemental dose, the epidural catheter was resited. A patient-controlled epidural analgesia (PCEA) pump was attached 30 minutes after the last loading dose, which was established with a mixture of 0.1% ropivacaine plus 0.5 μg/mL sufentanil and programmed to deliver 8 mL boluses with a lockout interval of 30 minutes, a background infusion of 8 ml/h, and maximum dose of 24 ml/h. The PCEA pump was discontinued when the cervix was dilated to 10 cm. Mothers and babies were encouraged to stay in the same room and to start breastfeeding as early as possible. During labor analgesia, continuous fetal heart rate and ECG monitoring and analgesia recording were performed.

### Data collection

Before the obstetric anesthesia clinic was opened, the baseline data were collected in the obstetric clinic, and after the clinic worked, the baseline data was collected there. All eligible parturients were asked to complete a number of paper questionnaires, and to complete them independently to avoid the interference from family members. Demographic data included maternal age, height, weight, educational background, family monthly incomes, stable occupation, marital status, history of adverse pregnancy, and gravity. Social psychological variables data included medical payment, family preference for the baby’s gender. Maternal comorbidities (hypertension, gestational diabetess, thyroid disease, anemia). Parturients were asked to fill the EPDS form. It is a self-report questionnaire consisting of 10 items; each item is rated from 0 to 3, denoting increasing severity of symptoms, with the maximum score being 30. The EPDS has been widely used as a screening tool for both prenatal and postpartum depression. The Chinese version had been validated and a cut-off score of 9/10 was recommended for screening depression [34]. Participants were also asked to fill the following forms to assess the levels of anxiety (using the Zung Self-Rating Anxiety Scale, SAS) and fear of childbirth (using Wijma Delivery Expectancy Questionnaire, W-DEQ). Reassessed antenatal anxiety and fear of childbirth when the women entered the delivery room.

Intrapartum data included the duration of labor (starting with regular contraction and ending with placenta delivery), mode of delivery, perineal lacerations (none; first, second, third, or fourth degree), and the amount of bleeding. For parturients who received LEA, their NRS pain scores were assessed respectively, before labor analgesia, 15 and 30 minutes after analgesia, and at full cervical dilation. Complications including dural perforation and supine hypotension syndrome were recorded. For those who did not receive LEA, their NRS pain scores were assessed at 2 cm of cervical dilation and at full cervical dilation. Neonatal data included sex, body weight, apgar Scores at 1, and 10 minutes after delivery, and admission to the neonatal ward or neonatal intensive care unit.

The pre-discharge follow-up before discharge was performed at second day postpartum. Parturients were asked to rate their satisfaction with childbirth course, pain management and the overall perinatal care. Participants responded using a five-point scale: 5 = very satisfied 4 = satisfied, 3 = neither satisfied nor dissatisfied, 2 = dissatisfied, or 1 = very dissatisfied. The mode of baby feeding (breast feeding, mixed feeding, or formula feeding), Willingness to have a second child, who the parturients live with after giving birth,and NRS score were recorded. Follow-up visits at 1, 2 and 6 weeks after delivery were conducted by telephone interview and the follow-up included EPDS scores, NRS scores and infant feeding practices. The primary endpoint was the presence or absence of PPD (defined as an EPDS score ≥ 10) at 6 weeks after delivery.

### Sample size estimation

According to the published literature and our preliminary study, it was assumed that the incidence of postpartum depression was 25% in the nonmedicated parturients and 12.5% in the parturients who received anaesthesia clinic childbirth course combined with LEA. The calculated sample size that would provide 80% power to see this difference based on a 2-tailed significance level of 0.05 is about 600 participants. Considering an estimated attrition rate of 6%, the final sample size was 640 participants, 160 in each group. The sample size calculation was performed PASS 15.0 analysis program.

### Statistical strategies

Continuous variables are presented as mean ± standard deviation (SD) or median (IQR). Data were compared by One-Way ANOVA (parametric) / Kruskall - Wallis H (non-parametric) tests. Categorical variables are presented as number of patients (percentage). Data were analyzed with the use of χ^2^ test or Fisher exact test. All independent variables were assessed as univariate association with PPD. Variables that were significant in univariate analyses (*P* ≤ 0.1) or were considered clinically significant were checked for collinearity by using bivariate correlation analyses. Those that had little or no collinearity were modeled in multivariate logistic regression to determine the risk-adjusted predictors of PPD by using a backward (conditional) stepwise procedure. Two - sided *P* values < 0.05 were regarded as significant. Statistical analyses were performed with SPSS 23.0 software.

## Results

### Participant demographics and baseline variables

**Fig. 1:**
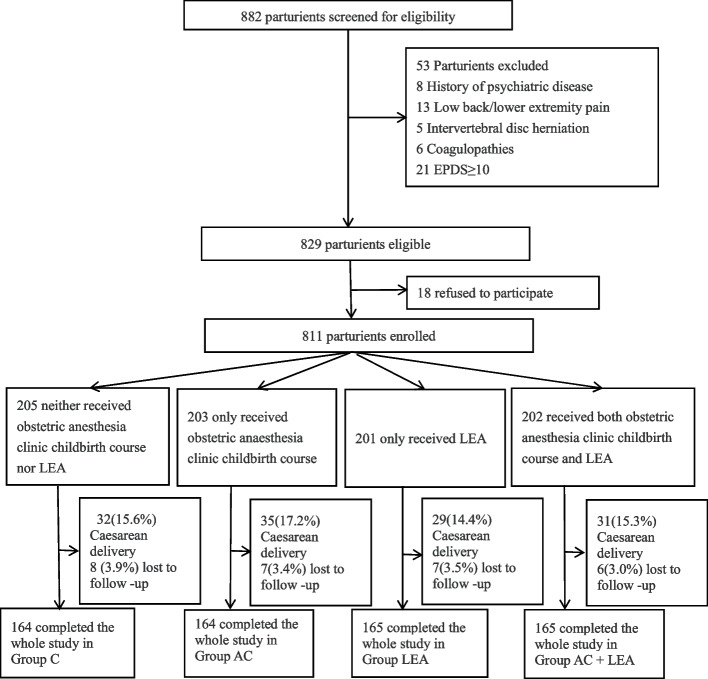
Flow chart of the study

From October 21, 2020 to December 31, 2021, 882 parturients were screened. Of these, 829 were eligible and 811 were enrolled in the study. During the delivery period, 120 parturients changed their minds and chose to have a cesarean section. During the postpartum period, 36 were lost to follow-up. At last, 655 parturients completed the study. There was no difference in the proportion of women lost to follow-up among groups (Fig. 1).

No significant differences in parturients demographics among the four groups were found, including age, body mass index, educational background, family monthly incomes, stable occupation, marital status, planned pregnancy, history of adverse pregnancy, gravidity, medical payment, maternal comorbidities and prenatal examination. Baseline characteristics of parturients who completed the study are shown in Table 1.

**Table 1:** Baseline characteristics of women who completed the study

Variables	Group C (*n* = 164, %)	Group AC (*n* = 161, %)	Group LEA (*n* = 165, %)	Group AC + LEA (*n* = 165, %)	*P*
Age (year)	25.4 ± 3.6	26.0 ± 3.3	25.9 ± 3.2	25.9 ± 3.1	0.341
Body mass index (kg/m^2^)	27.6 ± 1.8	27.8 ± 2.0	27.7 ± 2.1	28.0 ± 2.0	0.426
Educational background					0.493
Middle school	24 (12.2%)	15 (9.3%)	17 (10.3%)	16 (9.7%)	
High school	83 (50.6%)	84 (52.2%)	85 (51.5%)	80 (48.5%)	
Bachelor or post-graduate	61 (37.2%)	62 (38.5%)	63 (38.2%)	69 (41.8%)	
Family monthly incomes (RMB, Yuan)					0.056
≤ 5000	7 (4.3%)	6 (3.7%)	3 (1.8%)	3 (1.8%)	
5001–10,000	72 (43.9%)	65 (40.4%)	60 (36.4%)	49 (29.7%)	
≥ 10,000	85 (51.8%)	90 (55.9%)	102 (61.8%)	113 (68.5%)	
With stable occupation	112 (68.3%)	118 (73.3%)	126 (76.4%)	131 (79.4%)	0.117
Place of residence					0.122
City	59 (36.0%)	71 (44.1%)	78 (47.3%)	85 (51.5%)	
Township	50 (30.5%)	48 (29.8%)	42 (25.5%)	84 (26.7%)	
Rural	55 (33.5%)	42 (26.1%)	45 (27.2%)	36 (21.8%)	
Marital status					0.935
Married	158 (96.3%)	156 (96.9%)	161 (97.6%)	160 (97.0%)	
Single or divorced	6 (3.7%)	5 (3.1%)	4 (2.4%)	5 (3.0%)	
Planned pregnancy	108 (65.9%)	112 (69.6%)	114 (69.1%)	118 (71.5%)	0.742
History of adverse pregnancy	32 (19.5%)	35 (21.7%)	33 (20%)	30 (18.2%)	0.880
Gravidity					0.358
1	102 (62.2%)	105 (65.2%)	110 (66.7%)	109 (66.1%)	
2	45 (27.4%)	43 (26.7%)	37 (22.4%)	34 (20.6%)	
3	11 (6.7%)	11 (6.8%)	14 (8.5%)	12 (7.3%)	
4	6 (3.7%)	2 (1.2%)	4 (2.4%)	10 (6.1%)	
Attend an obstetric delivery course	12 (7.3%)	18 (11.2%)	13 (7.9%)	19 (12.7%)	0.286
smoking during pregnancy	2 (1.2%)	1 (0.6%)	3 (1.8%)	2 (1.2%)	0.811
drinking during pregnancy	5 (3.0%)	4 (2.5%)	7 (4.2%)	6 (3.6%)	0.833
Medical payment					0.198
Medical insurance	113 (54.9%)	107 (57.1%)	121 (68.5%)	126 (76.4%)	
Self-paying	51 (45.1%)	54 (42.9%)	44 (31.5%)	39 (23.6%)	
Maternal comorbidities					0.343
Hypertension	0 (0.0%)	1 (0.6%)	2 (1.2%)	2 (1.2%)	
Diabetes	20 (12.2%)	26 (16.1%)	25 (15.2%)	27 (16.4%)	
Hypothyroidism	10 (6.1%)	25 (15.5%)	15 (9.1%)	17 (10.3%)	
Anemia	8 (4.9%)	6 (3.7%)	11 (6.7%)	13 (7.9%)	
EPDS score	6 (5, 7)	6 (5, 7)	6 (5, 7)	6 (5, 7)	0.152

Data are presented as mean ± SD, number of patients (percentage);

*EPDS* Edinburgh Postnatal Depression Scale

### SAS score and W-DEQ score

There were no significant difference in the SAS score between the clinic and the delivery room. The SAS scores in the delivery room in the Group AC and the Group AC + LEA were lower than those in the clinic (Table 2). The results of the W-DEQ score comparison were consistent with the results of the SAS score comparison (Table 3).

**Table 2:** Comparison of SAS score between delivery room and clinic

	Group C (*n* = 164, %)	Group AC (*n* = 161, %)	Group LEA (*n* = 165, %)	Group AC + LEA (*n* = 165, %)	*P*
SAS score in the clinic	33 (30, 41)	37 (34,41)	36 (31, 42)	38 (35, 43)	0.112
SAS score in the delivery room	32 (30, 39)	33 (30,40)	35 (31, 40)	32 (30, 37)	0.074
*P*	0.069	< 0.001	0.198	< 0.001	

Data are presented as median (TQR), SAS, Zung Self-Rating Anxiety Scale

**Table 3:** Comparison of W-DEQ score in clinic and delivery room

	Group C (*n* = 164, %)	Group AC (*n* = 161, %)	Group LEA (*n* = 165, %)	Group AC + LEA (*n* = 165, %)	*P*
W-DEQ score in the clinic	71 (66, 74)	71 (65, 74)	70 (66, 73)	72 (68, 74)	0.337
W-DEQ score in the delivery room	70 (65, 75)	68 (65, 73)	70 (66, 73)	69 (61, 73)	0.121
*P*	0.142	< 0.001	0.100	< 0.001	

Data are presented as median (TQR), *W-DEQ* Wijma Delivery Expectancy/Experience Questionnaire

### Intrapartum variables

Intrapartum variables in the four groups were shown in Table 4. The NRS pain scores at 10-cm cervical dilation, compared to the Group C and Group AC, was significantly lower than in the Group LEA and group AC + LEA (*P* <  0.001).

**Table 4:** Intrapartum variables of women who completed the study

	Groupe C (*n* = 164)	Group AC (*n* = 161)	Groupe LEA (*n* = 165)	Groupe AO + LEA (*n* = 165)	*P*
NRS pain score					
at 2-cm cervical dilation	7 (7, 8)	7 (7, 8)	7 (7, 8)	7 (7, 8)	0.393
15 minutes after analgesia	–	–	3 (2, 4)	3 (2, 4)	–
30 minutes after analgesia	–	–	2 (2, 3)	2 (2, 3)	–
at 10-cm cervical dilation	10 (8, 10)	9 (7, 10)	4 (4, 6)*^#^	4 (4, 6)*^#^	< 0.001
duration of labor (min)					
First stage (min)	350 (297, 418)	343 (302, 407)	360 (296, 436)	373 (300, 460)	0.226
Second stage (min)	39 (30, 45)	39 (31, 49)	40 (33, 51)	39 (32, 50)	0.291
Third stage (min)	8 ± 2	7 ± 2	7 ± 2	8 ± 2	0.596
Mode of delivery					0.510
Spontaneous delivery	159 (97%)	157 (97.5%)	158 (95.8%)	156 (94.5%)	
Assisted vaginal - vacuum	5 (3%)	4 (2.5%)	7 (4.2%)	9 (5.5%)	
Lateral episiotomy	70 (42.7%)	66 (41.0%)	61 (37.0%)	63 (38.2%)	0.708
Estimated blood loss (mL)	200 (150, 250)	200 (150, 250)	200 (150, 270)	200 (170, 250)	0.273

Data are presented as mean ± SD, number (%) or median (TQR)

Numeric rating scale (NRS), where 0 = no pain and 10 = the worst pain

Compared with Group C, * *P* <  0.05; compared with Group AC, ^#^*P* <  0.05

### Neonatal demographics

The neonatal demographics of the four groups are shown in Table 5. No significant differences were observed in neonatal variables of the four groups.

**Table 5:** Neonatal demographics

	Groupe C (*n* = 164)	Group AC (*n* = 161)	Groupe LEA (*n* = 165)	Groupe AO + LEA (*n* = 165)	*P*
Neonatal gender					0.983
male	83 (50.6%)	83 (51.6%)	87 (52.7%)	84 (50.9%)	
female	81 (49.4%)	78 (48.4%)	78 (47.3%)	81 (49.1%)	
Neonatal body weight (kg)	3.23 ± 0.37	3.22 ± 0.36	3.30 ± 0.32	3.26 ± 0.36	0.170
Apgar score after birth					
One-minute	10 (10, 10)	10 (10, 10)	10 (10, 10)	10 (10, 10)	0.132
Five-minute	10 (10, 10)	10 (10, 10)	10 (10, 10)	10 (10, 10)	0.198
Baby’s gender consistent with family’s preference	128 (77.1%)	120 (74.5%)	116 (70.3%)	119 (72.1%)	0.531
Admission to neonatal ward after birth	16 (9.8%)	14 (8.7%)	13 (7.9%)	11 (6.7%)	0.772

Data are presented as mean ± SD, number (%) or median (TQR)

### Postpartum outcomes

At 6 weeks after delivery, developed PPD. The incidence of PPD and EPDS scores were significantly lower in Group AC + LEA than in Group C. (Table 6).

**Table 6:** Comparison of postpartum outcomes among the groups

	Groupe C (*n* = 164)	Group AC (*n* = 161)	Groupe LEA (*n* = 165)	Groupe AO + LEA (*n* = 165)	*P*
Second day postpartum					
NRS of pain (score)	4 (3, 4)	4 (3, 4)	3 (3, 3)*^#^	3 (3, 3)*^#^	< 0.001
Breast-feeding	139 (84.8%)	142 (88.2%)	144 (87.3%)	146 (88.5%)	0.740
Labor pain management satisfaction	2 (2, 3)	2 (2, 3)	4 (3, 4)*^#^	4 (4, 4)*^#^	< 0.001
Obstetric anesthesia clinic satisfaction	–	4 (4,5)	–	4 (4, 5)	
Perinatal care satisfaction	3 (2, 3)	3 (3, 4)*	3 (3, 4)*	4 (3, 4)*^#&^	< 0.001
Willingness to have a second child	51 (31.1%)	53 (32.9%)	67 (41.6%)*^#^	89 (53.9%)*^#&^	< 0.001
Postdelivery living					0.736
Mother and husband	71 (43.3%)	60 (37.3%)	74 (44.8%)	62 (37.6%)	
Mother-in-law and husband	69 (42.1%)	79 (49.1%)	65 (40.0%)	72 (43.6%)	
Confinement centre	24 (14.6%)	22 (13.7%)	25 (15.2%)	31 (18.8%)	
A week postpartum					
NRS of pain (score)	2 (1, 2)	2 (1, 2)	2 (1, 2)	2 (1, 2)	0.326
EPDS score	7 (5, 8)	6 (6, 7)	6 (6, 7)	6 (5, 8)	0.309
Breast-feeding	145 (88.4%)	147 (91.3%)	150 (90.9%)	149 (90.3%)	0.822
Two weeks postpartum					
NRS of pain (score)	2 (1, 2)	2 (1, 2)	2 (1, 2)	2 (1, 2)	0.326
EPDS score	7 (5, 8)	6 (6, 7)	6 (6, 7)	6 (5, 8)	0.309
Breast-feeding	145 (88.4%)	147 (91.3%)	150 (90.9%)	149 (90.3%)	0.822
Six weeks postpartum					
NRS of pain (score)	0 (0, 0)	0 (0, 1)	0 (0, 1)	0 (0, 1)	0.471
NRS pain score > 3	13 (7.9%)	11 (6.8%)	12 (7.3%)	11 (6.7%)	0.965
Exclusive breast-feeding	106 (64.6%)	113 (70.2%)	106 (65.0%)	110 (66.7%)	0.706
EPDS score	7 (5, 11)	6 (5, 7)	6 (5, 8)	6 (5, 7)*	0.023
Incidence of 6-week PPD^a^	44 (26.8%)	32 (19.9%)	28 (17.0%)	20 (12.1%)*	0.014
Baby readmitted to hospital	3 (1.8%)	2 (1.2%)	2 (1.2%)	3 (1.8%)	0.938
Twelve weeks postpartum					
NRS of pain (score)	0 (0, 0)	0 (0, 0)	0 (0, 0)	0 (0, 0)	0.87
NRS pain score > 3	6 (3.7%)	5 (3.1%)	5 (3.0%)	3 (1.8%)	0.79
Exclusive breast-feeding	105 (64 %)	97 (60.2%)	104 (63.0%)	106 (64.2%)	0.87
EPDS score	4 (3, 5)	4 (3, 5)	4 (3, 5)	4 (3, 5)	0.11

Data are presented as mean ± SD, number (%) or median (TQR)

*NRS* numeric rating scale where 0 = no pain and 10 = the worst pain

*EPDS* Edinburgh Postnatal Depression Scale;

^a^Defined as EPDS score ≥ 10;Compared with Group C, * *P* < 0.05; compared with Group AC, ^#^*P* < 0.05; compared with Group LEA, ^&^ *P* < 0.05

On the second day after delivery, Group LEA and AC + LEA groups had lower NRS scores than Group C and AC (*P* <  0.001). Satisfaction with labour pain management was higher in Group LEA and AC + LEA than in Group C and AC (*P* <  0.001). Perinatal care satisfaction in the group C was significantly lower than that of the other three groups (*P* <  0.001), while the satisfaction of perinatal care in the Group AC and Group LEA was lower than that of the Group AC + LEA (*P* <  0.001).

### Association between obstetric anaesthesia clinic childbirth course combined with LEA and PPD at 6 weeks postpartum

Univariate logistic regression analysis showed that 9 variables were selected as candidates, including SAS score in the delivery room, W-DEQ score in the delivery room, NRS pain score at 10-cm cervical dilation, admission to neonatal ward after birth, postdelivery living, EPDS score at 1 week postpartum, subgroup variables, satisfaction with labor pain management, and satisfaction with perinatal care. These factors (*P* < 0.1) were considered for the multivariate logistic regression by using a backward (conditional) stepwise procedure.

**Fig. 2:**
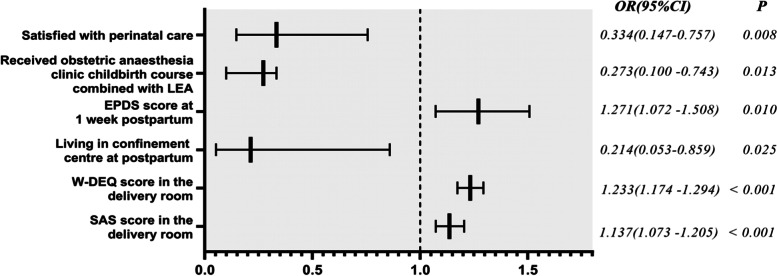
Forest plot of risk factors for PPD at 6-week postpartum

The regression results of multivariate logistics are shown in Table 7 and Fig. 2. Received obstetric anaesthesia clinic childbirth course combined with LEA is associated with a decreased risk of PPD. Multivariate logistic regression analysis identifies 5 other independent factors for PPD, including SAS score in the delivery room, W-DEQ score in the delivery room, living in confinement centre at postpartum, EPDS score at 1 week postpartum, and satisfaction with perinatal care.

**Table 7:** Variables associated with the occurrence of postpartum depression at 6 weeks

Factors	OR	95% CI	*P*
SAS score in the delivery room	1.137	1.073–1.205	< 0.001
W-DEQ score in the delivery room	1.233	1.174–1.294	< 0.001
Living in confinement centre at postpartum	0.214	0.053–0.859	0.025
EPDS score at 1 week postpartum	1.271	1.072–1.508	0.010
Received obstetric anaesthesia clinic childbirth course combined with LEA	0.273	0.100–0.743	0.013
Satisfied with perinatal care ^a^	0.334	0.147–0.757	0.008

OR, odds ratio; CI, confidence interval

*SAS* Zung Self-Rating Anxiety Scale, *W-DEQ* Wijma Delivery Expectation Questionnaire, *EPDS* Edinburgh Postpartum Depression Scale

^a^Evaluated by using a five-point scale (5 = very satisfied 4 = satisfied, 3 = neither satisfied nor dissatisfied, 2 = dissatisfied, or 1 = very dissatisfied). A score of 5 or 4 indicated satisfaction

## Discussion

Our study found that Nulliparous women with single term cephalic pregnancy who received obstetric anaesthesia clinic childbirth course combined with LEA was associated with a decreased risk of PPD at 6 weeks. While the incidence of 6-week PPD were significantly lower in Group AC + LEA than in Group C.

In China, the reported prevalence of PPD at 6 weeks ranges from 5.9 to 34.0% with an EPDS cutoff value of ≥10 [35, 36]. The prevalence of PPD in our study was 18.9% (124 / 655), which was within the reported range.

There is no universally accepted time point for postpartum depression screening [1, 37]. Depression in the postpartum period typically begins within 6 weeks after delivery but can occur any time during the first 12 months after delivery. 50–80% of women experience a brief episode of “baby blues”. It has also been suggested that screening for postpartum depression should not be performed in the first few days after delivery, as symptoms are not yet fully developed and may be confused with “baby blues”. However, a growing body of research suggests that high prenatal or perinatal depression scores are strong predictors of PPD [38]. Therefore, women with prenatal EPDS score ≥ 10 were excluded from this study to reduce confounding factors and sample bias, and were followed up with EPDS scores at 1, 2, 6 and weeks after delivery. The diagnosis of PPD was based on the 6-week EPDS score. We also found that high EPDS score at 1 weeks postpartum is independent predictor of PPD.

This observational cohort study shows that the use of LEA is not associated with the risk of postnatal depressive symptoms at 6-week postpartum. Our results are consistent with those of other studies in the literature [25, 39, 40]. Notably, our findings are in contrast to other studies examining the risk of LEA and PPD. The reasons for the contradictory results may be related to the following points. First, the etiology of PPD is multifactorial, labor pain is only one of the factors, and may not be the most important factor, and the reduction of pain is not enough to change the incidence of PPD. Second, some studies did not address whether LEA-naive pregnant women received other forms of pain relief, such as local anesthesia, anesthetics, nitrous oxide, non-pharmacological measures, etc. In our study, it was clearly stated that no other pain relief measures were given to pregnant women who did not use LEA. Third, these studieshave been criticized for subject selection bias, not appropriately controlling for measured confounding variables, not excluding pregnant women with antenatal depression, and excluding differential loss to follow-up. In our study, the follow-up dropout rates did not differ among the groups, and we excluded women who were transferred to cesarean section (a change in delivery mode may affect the maternal psychological state), which may also account for different results. So it is not clear whether LEA actually affects the risk of developing PPD.

Higher antenatal anxiety (SAS score), fear of Childbirth (W-DEQ score) were associated with an increased risk of PPD 6-week postpartum in our study. Women may experience fear and anxiety about childbirth due to lack of knowledge or misinformation about and negative perceptions about the birth processs [41]. Lack of knowledge and unpreparedness of women can lead to anxiety and complications, followed by ever-increasing medical interventions [41]. Evidence has also shown that participation in childbirth courses reduces anxiety about delivery and generates suitable response to pain [42]. Childbirth courses are designed to provide mothers with information to help them improve their ability to handle the childbirth process, improve pain relief, and positively impact the childbirth experience. Conventional childbirth courses are systematic courses that require multiple and long-term studies, so the participation rate of pregnant women is not high. Moreover, there is little detail in the courses about labor analgesia and evaluation of prenatal anxiety and fear of childbirth. Severe labor pain, anxiety before delivery and fear of delivery are all related to PPD. Therefore, we specifically designed an obstetric anesthesia clinic childbirth course led by an anesthesiologist. To our knowledge, this study is the first to determine the effect of the obstetric anesthesia clinic childbirth course combined with LEA to incidence of 6 - week PPD in women. Antenatal education has been reported to reduce fear of childbirth, increase maternal self - efficacy related to childbirth, and increase women’s satisfaction, which is consistent with our results [43]. It shows that our childbirth course is effective, can reduce anxiety and fear scores, and make it easier for pregnant women to cope with the psychological changes during childbirth. We did not find an association between receiving childbirth course and a reduction in PPD. It may be that the duration of education was too short and too little content compared to other study [44].

Pain management satisfaction in the Group LEA does not differ from that in Group AC + LEA, but perinatal care satisfaction in Group LEA is lower than that in group AC + LEA. Perinatal care satisfaction was also associated with PPD in this study. It may be that our childbirth course reduced prenatal anxiety and fear of childbirth for some women, while LEA significantly reduces pain during delivery for these women, leading to an increase in perinatal satisfaction and a decrease in PPD at 6 weeks. Pain, antenatal anxiety and childbirth fear, which are not important factors for PPD, reducing them at the same time can decrease the incidence of PPD, but changing only one of these factors does not work.

Our study found that living in confinement centre after childbirth is good for reducing PPD. In traditional Chinese culture, postnatal women are considered weakened, vulnerable to a yin and yang imbalance, and in need of special care. ‘Puerperium care’ is a traditional Chinese ritual that takes 30 days and involves physical and social prescriptions and taboos for women after childbirth. During the period of puerperium care, new mothers usually live with their parents or in-laws, or in postnatal care facilities [45]. Studies have reported that feelings of confinement affect postpartum depression [46]. Positive feelings include physical rehabilitation and recovery, opportunities to learn the care of and develop a bond with the newborn, and access to support and care. In contrast, negative feelings include taboos and too many rules, less freedom, and the conflict between traditional values and modern life, which places a burden on the mother’s psyche and increases the possibility of postpartum depression. However, as social life in China continues to change, more and more women are now living in nuclear families rather than big family. A large number of the new mothers who grow up in the one-child generation start to spend time with their mothers-in-law from the recovery period. During this fragile period, it is easy for conflicts to arise between the two parties, which may cause PPD. Postpartum care institutions provide maternal and baby medical care, dietary supplements, and other support measures so that the mother can get assistance and consultation at any time [47]. Huang reported that services provided by postpartum care institutions reduce the incidence of PPD [48].

This study has several limitations that may affect the ability to determine the true risk factors on PPD. Firstly, only Chinese nulliparous women were included, limiting the diversity of the cohort. Secondly, the diagnosis of PPD was not performed by psychiatrists. EPDS can be used by nonpsychiatric physicians to detect postpartum depression. EPDS is a self-report and user-friendly questionnaire that was used to evaluate PPD in the present study. However, it should be noted that EPDS is just a screening scale for PPD used by non-psychiatrists, and it is not a professional psychiatric diagnostic tool. EPDS has been used to assess PPD in many studies, mainly because it has an estimated sensitivity of 80% for the diagnosis of depression. Finally, it is not a randomized controlled study, but an observational study with many confounding factors.

This study found that there were statistical differences indicating that attending obstetric anesthesia courses and lumbar epidural anesthesia can reduce postpartum depression. However, the difference between the scores seems to be small. Due to the large sample size included in this study, it can be considered as a clinically significant difference. In addition, in the postpartum follow-up, although the difference between the two groups was slight, the difference was statistically significant, so it was considered that the impact of the intervention measures was different. The evidence of this study shows that attending obstetric anesthesia courses and performing lumbar epidural anesthesia at the same time can affect the psychological status of pregnant women. The long-term results can be further observed in subsequent studies, such as extending the observation period to 6 months or more, and observing the physical and neural development of the offspring.

## Conclusion

Our study shows that received obstetrics anaesthesia clinic childbirth course combined with LEA is associated with a decreased risk of PPD at 6 weeks. There are 5 other independent factors for PPD, including SAS score in the delivery room, W-DEQ score in the delivery room, living in confinement centre at postpartum, EPDS score at 1 week postpartum, and satisfaction with perinatal care. By setting up a joint obstetric anesthesia clinic, pregnant women can participate in the delivery courses of anesthesiologists, which can significantly improve the positive selection rate of pregnant women, and improve the service ability and social recognition of anesthesiologists.

## Data Availability

The datasets used and analyzed in this study is available from the corresponding author on reasonable request.
